# The VegPlate for Sports: A Plant-Based Food Guide for Athletes

**DOI:** 10.3390/nu15071746

**Published:** 2023-04-03

**Authors:** Luciana Baroni, Ettore Pelosi, Francesca Giampieri, Maurizio Battino

**Affiliations:** 1 Scientific Society for Vegetarian Nutrition, 30171 Venice, Italy; 2 Sport Nutrition Department, Multispecialistic Medical Center, CDC-Affidea, 10128 Turin, Italy; 3 PET/CT Nuclear Medicine Department, Irmet Affidea Center, 10135 Turin, Italy; 4 Research Group on Food, Nutritional Biochemistry and Health, Universidad Europea del Atlántico, 39011 Santander, Spain; 5 International Research Center for Food Nutrition and Safety, Jiangsu University, Zhenjiang 212013, China; 6 Department of Clinical Specialistic and Odontostomatological Sciences, University Politecnica delle Marche, 60121 Ancona, Italy

**Keywords:** VegPlate, sport, athlete, plant-based, vegetarian, vegan, lacto-ovo-vegetarian

## Abstract

Background: Nutrition strategies improve physiological and biochemical adaptation to training, facilitate more intense workouts, promote faster recoveries after a workout in anticipation of the next, and help to prepare for a race and maintain the body’s hydration status. Although vegetarianism (i.e., lacto-ovo and veganism) has become increasingly popular in recent years, the number of vegetarian athletes is not known, and no specific recommendations have been made for vegetarian dietary planning in sports. Well-planned diets are mandatory to obtain the best performance, and the available literature reports that those excluding all types of flesh foods (meat, poultry, game, and seafood) neither find advantages nor suffer from disadvantages, compared to omnivorous diets, for strength, anaerobic, or aerobic exercise performance; additionally, some benefits can be derived for general health. Methods: We conceived the VegPlate for Sports, a vegetarian food guide (VFG) based on the already-validated VegPlate facilitating method, designed according to the Italian dietary reference intakes (DRIs). Results: The VegPlate for Sports is suitable for men and women who are active in sports and adhere to a vegetarian (i.e., lacto-ovo and vegan) diet, and provides weight-based, adequate dietary planning. Conclusions: The VegPlate for Sports represents a practical tool for nutrition professionals and gives the possibility to plan diets based on energy, carbohydrate (CHO), and protein (PRO) necessities, from 50 to 90 Kg body weight (BW).

## 1. Introduction

“Exercise is King, while nutrition is Queen: put them together and you have a Kingdom!” (J. Lalanne [1]).

Vegetarian diets are plant-based diets in which any kind of animal flesh is excluded (meat, poultry, wildfowl, seafood, and their derivatives), and are based on a varied amount of plant foods. The presence of milk and eggs and of their derivatives distinguishes lacto-ovo-vegetarian diets from vegan diets. The plant foods included in a vegetarian diet are grains, legumes, vegetables, fruits, and nuts and seeds [2]. The reasons of this choice are ethical, health-related, and environmental ones [3].

### 1.1. Different Types of Diets Followed by Athletes

Athletes may follow various types of diets, depending on their specific goals and requirements. Each type of diet has its advantages and disadvantages, and it is important for athletes to choose a diet that aligns with their goals and needs.

Some popular diets among athletes include:

- High-carbohydrate diets: these are diets commonly followed by endurance athletes, as carbohydrates provide a quick source of energy for prolonged physical activity [4].
- High-protein diets: these diets are often followed by athletes who are looking to build muscle mass or recover from injury [5].
- Ketogenic diets: these are diets low in carbohydrates and high in fat, and are followed by some athletes to improve endurance and weight loss [6].
- Vegetarian (lacto-ovo-vegetarian and vegan diets): these diets eliminate animal products and can be usually followed for ethical, environmental, or health reasons [2].

### 1.2. Athletes’ Trend towards Vegetarian Diets

Available population statistic data show that, in 2019, 12% of European citizens followed a vegetarian diet [7]. Wirnitzer reports that 6% of the US population is vegetarian (lacto-ovo-vegetarian and vegan) and that about 10% of Europeans adhere to some type of vegetarian diet. These diets are popular mainly in younger generations. “As a consequence, it is very likely that there is no longer any social group or sports team without a vegan person or athlete. Unpublished data from our laboratory considering the NURMI Study (Step 1) show the prevalence of 35% vegans, 21% lacto-ovo-vegetarians and 44% omnivores starting in running events” [8].

The demand among athletes for plant-based diets represents a growing trend. A survey of over 300 athletes conducted by researchers at the University of Winchester found that 33% of respondents were interested in following a lacto-ovo-vegetarian or vegan diet, while another 29% were considering it [9]. Similarly, a survey of over 200 endurance athletes conducted by researchers at the University of Oxford found that 33% were lacto-ovo-vegetarian or vegan [10].

In recent years, more and more athletes, including famous professional athletes (e.g., Lewis Hamilton and Novak Đoković) declared they followed a vegetarian diet (lacto-ovo or vegan) [11], due to health, ethical, or environmental grounds [2,12,13]. Nevertheless, the exact global number of vegetarian athletes is not known.

### 1.3. Motivation of the Choice

There are several reasons why athletes may choose to follow a vegetarian diet, including:

- Health benefits: A vegan diet that is well-planned and adequate provides all the necessary nutrients for optimal health, including protein, iron, calcium, and vitamin B12 [2]. A diet rich in fruits, vegetables, whole grains, and legumes can also provide antioxidants and anti-inflammatory compounds that can help reduce inflammation and disease risk [14,15,16].
- Environmental sustainability: many athletes are concerned about the impact of their food choices on the environment, and a plant-based diet can be more environmentally sustainable than a diet that includes animal foods [17].
- Ethical concerns: some athletes may choose a vegan diet because of ethical concerns about the treatment of animals in the food industry [18,19].
- Improved athletic performance: There is some evidence suggesting that a plant-based diet might improve athletic performance and recovery time after exercise [2,8,15,20,21]. For example, a study published in the International Journal of Sport Nutrition and Exercise Metabolism found that a vegan diet was associated with better endurance performance in elite female runners [9].
- Weight management: A vegan diet can be an effective way to manage weight, which can be important for athletes in weight-dependent sports. Vegan diets tend to be lower in calories and fat, and higher in fiber, which can help promote satiety and weight loss [22,23].

### 1.4. Importance of Proper Nutrition for the Athlete

Athletes in all sports strive to adopt nutritional strategies that will enhance their mental and physical performance, while also supporting their overall wellbeing [19,21]. This drive lies at the heart of sport nutrition, which not only involves a healthy and appropriate nutrition plan to fuel the high energy demands of athletic activities, but also calls for specific nutrient intake before, during, and after training or competition. Moreover, maintaining a body weight (BW) that is appropriate for the specific sport being played is crucial [24,25,26,27,28].

Nutrition strategies improve physiological and biochemical adaptation to training, facilitate more intense workouts, promote faster recoveries after a workout in anticipation of the next, and help to prepare for a race and to maintain the body’s hydration status. To support the best athletic performance, diets should be based on BW and lean body mass and characterized by high carbohydrate (CHO) content (3–12 g/kg/day), moderate/high protein (PRO) content (1.2–2 g/kg/day), and 20–35% of balanced fats among saturated, monounsaturated, and polyunsaturated ones [24,25,26]. Well-planned diets are mandatory to obtain the best performance, and the available literature reports that those excluding all types of flesh foods (meat, poultry, game, and seafood) neither find advantages nor suffer from disadvantages, compared to omnivorous diets, for strength, anaerobic, or aerobic exercise performance [29,30].

Both observational and short-term intervention studies, where subjects consumed either vegetarian or nonvegetarian diets for several weeks, failed to uncover any significant differences in strength/power, in aerobic and anaerobic performance parameters [9,29,30].

The proper timing of carbohydrate (CHO), protein (PRO), and water intake, in relation to training and competition, helps to optimize performance, ensures adequate recovery, and fosters muscle protein synthesis [28]. Nevertheless, until now, the discussion on vegetarian diets and the body in sports has been also a matter of general health and disease prevention, apart from athletic performance [31].

With regard to general health, some athletes tend to develop atherosclerotic lesions and cardiovascular disease earlier than sedentary people. A 2017 study [32] found the presence of coronary plaques in 44% of a group of endurance athletes (specifically cyclists and runners), while in the control group of sedentary peers, the percentage was 22%. A 2009 German study had already found myocardial damage in 12% of a group of marathon runners over 50 years old, against 4% of controls chosen among sedentary peers [33]. A very recent study described that lifelong endurance athletes had more coronary plaques, including more noncalcified plaques in proximal segments, than fit and healthy individuals with a similarly low cardiovascular risk profile [34].

These studies failed to demonstrate what were the causes capable of accelerating coronary heart disease in athletes, namely whether these problems were due to sports activity or to the foods often used to support it. However, it is likely that nutrition plays an important role: if the supplementary energy is provided (even in part) by animal products, the saturated fats and cholesterol they contain, combined with the absence of plant antioxidants and fibers, end up contributing to the development of atherosclerosis [22].

It still remains to be determined whether the long-term consumption of a vegetarian diet can improve recovery, prevent inflammation, and mitigate oxidative damage that occurs with intense workouts [35], or if it can induce a plasma-alkalizing effect in order to buffer the increase acidity of intense exercise and, thus, enhance athletic performance [36].

It is important to ensure that a vegetarian diet includes a variety of nutrient- and calorie-dense foods, and that the athletes pay close attention to meeting their daily nutrient needs, especially for certain critical nutrients, such as vitamins B12 and D, iron, and omega-3 fatty acids [13,15,25,37,38,39].

Taking all of this into account, there are no specific reasons or scientific evidence to suggest that a vegetarian diet should be different in terms of quality and quantity, when compared to an omnivorous diet for an athlete [40,41]. As such, the basic principles of a vegetarian sports meal remain the same as those of a nonvegetarian meal, with the same need for timing, quality and quantity of macronutrient choices, hydration, and supplementation [25,38].

### 1.5. Usefulness of a Vegetarian Food Guide Specific for the Athlete

Some vegetarian food guides (VFG) have been proposed for adult vegetarians since 1997 [42,43], but to date, no VFG has been proposed for athletes.

For this reason, we conceived the VegPlate for Sports, a VFG aimed to provide a practical tool for nutrition professionals, intended to satisfy athletes’ choices and their nutritional needs [44,45].

The vegetarian diets obtained with the VegPlate for Sports meet all the standard criteria to be respected for defining such diet as “well-planned” and are, therefore, suitable to meet the athletes’ needs. These criteria are (a) including a wide variety of plant foods (grains, legumes, vegetables, fruits, and nuts and seeds (dairy products and eggs are considered optional for reaching the diet’s adequacy); (b) choosing fats of plant origin, while consuming good sources of n-3 fatty acids (flaxseeds, chia seeds, and walnuts); (c) including reliable sources of calcium, and paying attention to the status of both vitamin B12 and vitamin D [43].

The aim of this perspective paper is to present a practical vegetarian food guide, the VegPlate for Sports, which might allow nutrition professionals to plan a vegetarian diet for athletes, having the immediate possibility of checking its content in energy, macronutrients, as well as micronutrients.

## 2. Methods

We conceived a facilitating tool for nutrition professionals looking after vegetarian (i.e., lacto-ovo and vegan) athletes, the VegPlate for Sports.

In doing so, we referred to the VegPlate method, already published in its adaptations for adults, pregnant and breastfeeding women, and children [43,46], which divides the allowed foods in “food groups” and uses the “servings system”.

Criteria for food selection and serving size calculation have been described in detail in the VegPlate for adults [43].

### 2.1. The Food Groups of the VegPlate for Sports

As already described in the above-cited publication [43], the basic VegPlate includes 6 fundamental “food groups”, which must be present in the everyday diet in quantities that are functional to satisfy the nutritional needs of the subject. The 6 fundamental food groups of the VegPlate are (a) grains; (b) protein-rich foods; (c) vegetables; (d) fruits; (e) nuts and seeds; and (f) fats. For each food group, we selected the most representative plant foods from the Mediterranean tradition.

To meet nutritional adequacy, the VegPlate also includes 2 cross-sectional food groups:

(g) calcium-rich foods, formed by the foods richest in calcium of all food groups, excluding the group of fats;

(h) omega-3-rich foods, made up of foods rich in omega-3, located in the group of nuts and seeds, and in the fats group.

The VegPlate is completed by the indications relating to the adequate intake of vitamin B12 and vitamin D. In the diagram, they are placed at the center of the plate to underline their indispensable presence in any balanced diet.

### 2.2. The Servings System

The “servings system” is conceived to indicate the quantity of each food of the same food group that provides comparable quantities of energy and nutrients and allows varying the foods within the same group without the need for rigorous exchange lists. According to this approach, serving sizes for each item were calculated [43].

Table 1 lists the serving sizes of the different foods belonging to the same food group and indicates calcium-rich foods and omega-3-rich foods [43].

The nutritional composition of one average serving from each group was calculated, according to Italian and USDA Food Databases [47,48]. Table 2 shows the energy, nutrient, fiber, and water composition of one average serving of each food group.

One serving of calcium-rich foods provides an average of 125 mg of calcium. One serving of omega-3-rich foods provides an average of 2.5 g of alpha-linolenic acid (ALA).

### 2.3. Adaptations of the VegPlate for Sports

To obtain the VegPlate for Sports, the original VegPlate method [43] received the necessary adaptations to meet the nutritional needs of the athlete.

In planning the adaptations of the VegPlate for Sports, we first reduced fiber as much as possible, mainly choosing nonwhole grains and limiting the number of servings to a maximum of 5 for vegetables and fruits.

A further reduction in fiber can be obtained from the centrifugation of fruits (5 whole servings provide approximately 12.5 g of fiber) and vegetables (5 whole servings provide approximately 11.5 g of fiber).

Given the high calorie intake and the wide variety of foods consumed, it was also unnecessary to assign a minimum number of servings of calcium-rich foods, since calcium needs are satisfied from the variety of the different foods.

However, the recommendation of consuming at least 2 servings of omega-3-rich foods is still valid (1 serving corresponds to 5 g of flaxseed oil, 10 g of flax seeds, 15 g of chia seeds, and 30 g of walnuts, as shown in Table 1).

Regarding the status of vitamins D and B12, we have already published our indications for the correction of any deficiency and the maintenance of an adequate status of the two vitamins in pregnant and breastfeeding women [49]. Since athletes’ needs for the two vitamins are no different from those of adults, and those of adults are equal or inferior to those of pregnant and breastfeeding women, it is possible to refer to these indications for athletes of both sexes.

## 3. Results

The average nutritional composition of one serving from each group was used to determine the daily number of servings to consume from each group in order to satisfy the Italian DRIs [44] for calorie requirements ranging from 1800 to 3200 kcal, WHICH CAN BE referred to a BW from 50 to 90 Kg. This nutritional composition also meets the USDA DRIs [45].

For each food group, the quantity of food of that group to be taken in relation to the calorie requirement, expressed as number of servings, was obtained.

Starting from an ideal BW range of 50–90 kg for both sexes, we calculated [50]:

(a) the basal metabolic rate increased by 30% (BMR × 1.3), according to which we created the “Main VegPlate”, which will provide the athlete with energy and nutrients for the main meals of the day.

We also calculated the intakes relating to the athlete’s phases of greatest physical activity. To satisfy the specific nutritional needs of the athlete in the two phases relating to the athletic performance (pre- and post-), two “small plates” were designed, distinct for each of the two phases, the “pre-” and “post-” VegPlates, which were placed alongside the Main VegPlate in the respective situations.

(b) the preworkout plate, the “pre-VegPlate”, included 1.5 g/kg of CHO, mostly slow-released, and 0.3 g/kg of PRO.

(c) the postworkout plate, the “post-VegPlate”, included 1.0 g/kg of CHO, mostly simple, and 0.3 g/kg of PRO.

The number of servings for each of the three plates, according to the respective BW, is summarized in Table 3, while Table 4 shows the nutritional composition (energy, fiber, and nutrients) of the serving patterns proposed in Table 3 [47,48].

Thanks to the servings system, it is possible to vary the foods in the diet using different foods from the same group, as indicated in Table 1.

Of course, this is a simplification that allows us to give an example of the food plan for each of the different BW categories considered, showing the simplicity and practicality of the method; anyway, it will then be the nutrition professional who draws up the food plan on the basis of the anthropometric characteristics, as well as the energy needs estimated on the basis of the sport practiced and the planned training hours.

Table 5 shows the percentage of calories provided by the three macronutrients out of the plan’s total calories.

**Figure 1:**
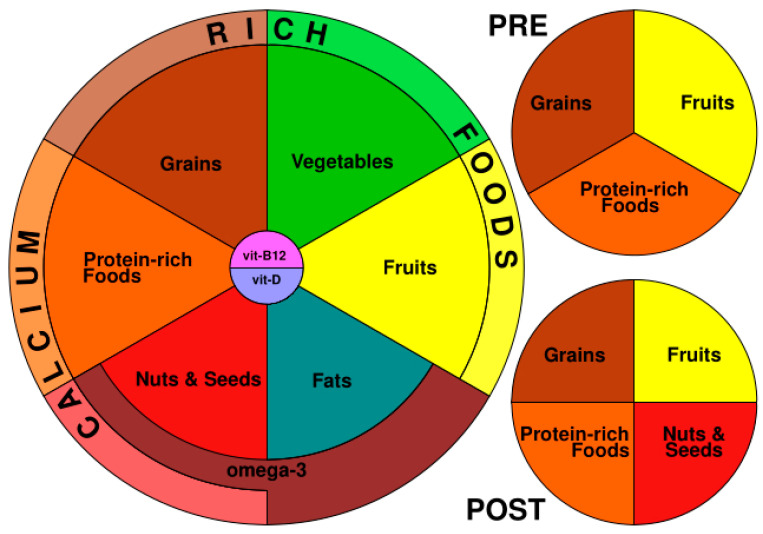
The VegPlate for Sports.

Finally, Figure 1 shows the diagram of the VegPlate for Sports: the main VegPlate, which must be accompanied by the two smaller plates, the pre- and post- VegPlate.

Since the quantities vary according to BW, the areas of the segments are representative only of the food groups composing the three plates, but not of the quantities; for this reason, all the segments have the same dimensions, and the quantities should be checked in Table 3.

## 4. Discussion

Regarding the lack of epidemiological data on the percentage of athletes following a vegetarian diet in Italy (and Europe), it is important to note that vegetarianism (i.e., lacto-ovo and veganism) has become increasingly popular in recent years. However, it is still unclear how many athletes in Italy and Europe follow a lacto-ovo-vegetarian or vegan diet, and how this may impact their performance [8].

The need for a food guide on lacto-ovo-vegetarian and vegan diets for athletes is rooted in the fact that athletes’ diets require careful planning to ensure that they are receiving all the nutrients required for optimal performance and recovery [2,8]. A dedicated food guide can help athletes understand how to balance their macronutrient and micronutrient intake to meet their specific needs and can also provide advice on food choices and meal planning.

As already highlighted, there is no conclusive evidence, to date, that vegetarian diets (i.e., lacto-ovo and vegan) represent a choice that improves athletic performance. However, it is also true that there is no conclusive evidence to support any disadvantages [51].

To support the best athletic performance, a combination of appropriate nutrition and the right workout plan will lead to the best results; all diets should be based on BW and lean mass and adjusted daily to the planned workout, focusing on CHO content (3–12 g/kg/day), PRO content (1.2–2 g/kg/day), and a balance of fats, including saturated, monounsaturated, and polyunsaturated ones [24,25,26].

Having access to simple tools that allows evaluating the energy content and nutritional values of the dietary plans is of great help when it is necessary to modify diets with the production of different menus with varying CHO and PRO content on a daily or continuous basis [29,43].

For plant-based nutrition, this is even more useful, considering the criticality related to the intake of fiber and some micronutrients, as well as the distrust that still exists towards vegetarian diets adopted by athletes. Nevertheless, the growing number of individuals choosing a plant-based diet, and the push towards environmental impact becoming increasingly oriented toward this direction, makes it necessary that nutritional professionals acquire the competence to correctly and easily plan a sports menu; we try to implement this with the VegPlate [43].

The schemes presented in the tables in this article are based on an estimated nutrient requirement (CHO about 55–60%, PRO about 15%, and LIP ≤ 35%), but primarily serve to familiarize the professional with the amount of food, expressed as number of servings for each food group, of the diet plan proposed by VegPlate, and to facilitate its use. In fact, it is the estimation of the assumed nutrient requirement, along with the grams/kg of CHO and PRO, that will lead to the final plan, using a few simple steps on a daily basis [43].

Table 1 and Table 2 aid in the comprehension of this system and explain the characteristics of the six different groups of foods (grains, protein-rich foods, vegetables, fruits, nut and seeds, and fats).

The dietary plan for athletes is primarily centered around the time of their workouts; the key meals in this plan are the preworkout meal (which should contain at least 1.5 g/kg of CHO and 0.3 g/kg of PRO) and the postworkout meal or meals (which should contain 1 g/kg of CHO and 0.3 g/kg of PRO, and can be repeated multiple times, if another workout is planned later in the day).

The preworkout meal is characterized by the presence of CHO with a moderate glycemic index; therefore, more fiber can be consumed if consumed 2 to 6 h before the workout [28,52]. The postworkout meal/meals, on the other hand, involves CHO with a high glycemic index, i.e., simple sugars to take advantage of the metabolically favorable moment, quickly replenishing the muscle’s glycogen stores, and repairing and adapting its protein components (metabolic window) [28,52,53]. They can be repeated every hour to refill muscle and hepatic glycogen stores depleted during training or competition [28,41].

### 4.1. Energy

The most crucial aspect in a sports diet is the energy intake; satisfying the energy requirement is a nutritional priority for all athletes [4,5,25,54]. An inadequate energy intake cancels the benefits of training, hinders performance, and can lead to health complications such as a loss of muscle mass and/or bone density, as well as an increased risk of overtraining, injuries, and illnesses [25,40]. Energy requirements vary among athletes depending on their sport, its intensity, and the periodic training activities they engage in (which can change from day to day and throughout the season). Other factors that impact energy requirement include gender, age, and body composition [25,54].

### 4.2. Carbohydrates

The role of CHO in the diet of athletes is also crucial. They are the main macronutrient (and ergogenic aid) along with PRO and water, and, as recommended by all experts, should represent the largest percentage of energy intake. The ingestion of CHO is essential in order to perform at optimal levels during any moderate-to-high-intensity training sessions that last longer than 90 min, or during intermittent activities typical of many team sports or certain types of training [4,25].

CHO is also necessary after exercise to restore muscle and liver glycogen levels, and to ensure adequate adaptation to training. The amount of CHO that active vegetarians need to consume varies depending on the sport, intensity, and BW.

The current recommendations for CHO are 5–10 g of CHO/kg BW/day for most athletes who perform moderate-to-high-intensity sports for approximately 1–3 h/day [25]. Lower intakes of 3–5 g/kg BW are suggested for athletes who perform low-intensity or skill-based training, while higher values of 8–12 g/kg BW are recommended during extreme-endurance training [4,25].

Although a typical vegetarian diet is rich in CHO, it is important to stress its role and remember its requirement, especially considering the recent popularity of low-CHO diets (KETO and the very-low-CHO diet VLCD), which are also attractive to some vegetarian athletes [55,56,57,58].

During long-lasting training sessions (longer than 90 min), vegetarian athletes, like all others, should be encouraged to consume simple CHO, up to 60–90 g/h, depending on the total duration [25,28,59]. This allows for a higher training intensity for longer periods of time, and favors recovery and adaptation [54,59,60].

Using the VegPlate system, “grains” and “fruits” represent the two food groups that allow manipulations of CHOs, by varying the number of servings (considering both starch and sugar; grains: 20.43 g and 1.45 g, respectively; fruit: 0.23 g and 14.81 g, respectively); CHOs are present even in protein-rich food, but only 5.08 g and 1.4 g of starch and sugar, respectively, are present. Grains are more useful for preworkout meals, together with protein-rich foods, while it is fruit for postworkout meals.

### 4.3. Protein

Emerging research on athletes’ PRO requirements suggests that dietary PROs “interact” with exercise by providing not only a substrate for the synthesis of contractile, structural, and metabolic proteins, but also for the stimulation of muscle protein synthesis [5,25]. Consuming a variety of plant protein sources throughout the day ensures that all essential amino acids are obtained as long as energy needs are met [39].

The PRO requirement, such as that of CHO, varies depending on the level of training and type of activity [61]:

- An athlete undergoing intense training needs more PRO than a person who is recreationally active and exercises moderately and only a few days a week;
- Endurance sports and strength sports have different requirements (they are greater for strength sports, which can reach 2 g/kg/day of BW).

Numerous studies on the diets of vegetarian endurance athletes have shown that they are meeting their recommended daily (RDA) PRO intake [9,38].

The RDA of 0.8 g of PRO per kilogram of BW per day is able to meet the needs of individuals who exercise at light-to-moderate intensity during the week [62]. However, considering the possible interference of fiber, phytates, and trypsin inhibitors with the absorption of amino acids contained in plant-based foods (lower bioavailability), for vegetarian diets, the needs may have to be increased by 10% [63], and for the nonathletic vegetarian population, values up to 1 g/kg/day have been suggested.

Protein digestibility is evaluated based on indices such as the protein-digestibility-corrected amino acid score (PDCAAS) and the digestible indispensable amino acid score (DIAAS); both show a higher score for animal proteins compared to those of plant origin. For example, rice, peas, and hemp are rated as lower than animal proteins in both scales, while soy proteins have a PDCAAS score identical to that of whey but are lower for the DIAAS [64,65,66].

The current recommendation on PRO intake for athletes of 1.2–2.0 g of PRO per kilogram of BW per day [25] is considered sufficient to support metabolic adaptation, repair, remodeling, and protein turnover. Some authors support the idea that for vegetarians, this level should be increased (up to 1.8/2.7 g per kilogram); however, recent papers question these results by proposing a daily intake threshold of 1.6 g/kg for complete essential amino acid profile proteins [67].

In a recent study, performed on healthy young adults consuming high-protein omnivorous or vegan diets, the different protein sources supported comparable rested and exercised daily myofibrillar protein synthesis rates in prolonged high-volume resistance training [68].

It is also important to note that, contrary to what was once believed, vegetarians do not need to consume specific combinations of plant proteins in each meal, but should consume a variety of protein sources distributed throughout the 24 h [2,69]. An exception may be considered for the postworkout period for athletes who perform intense strength training: in this phase, to support and optimize muscle protein synthesis, it is useful to “provide” leucine and about 10 g of essential amino acids [5,25]. Many plant proteins, including legumes, are rich in leucine, although not as bioavailable as those found in whey proteins. Additionally, the usual culinary combinations of proteins such as beans and rice, beans and nuts/seeds (e.g., in hummus), or a peanut butter sandwich are complementary [69]. The protein content of the diet can be manipulated by ADJIUSTING the NUMBER OF servings of protein-rich foods, each one providing 9.5 g of PRO.

### 4.4. Fats

Regarding fats, the guidelines for their intake for athletes should align with those issued by the public health system, and be personalized based on training goals and body composition [25]. Dietary fats are necessary to provide energy, cellular membrane structures and components, and essential fatty acids, and to promote the absorption of fat-soluble vitamins [70]. Fat stored within muscle cells and adipocytes is used as a substrate during moderate-intensity, prolonged exercise and, in general, during low-intensity activities [71]. Therefore, sources of “good” fats should be emphasized to optimize and meet recommendations for their intake; the intake of saturated fatty acids should be limited to less than 10% of total energy content [72].

In addition, particular attention should be paid to the consumption of omega-3 fatty acids that are important for maintaining heart health and reducing inflammation. Plant sources of omega-3 fatty acids include flaxseeds, chia seeds, and walnuts, and other promising sources [73,74,75,76,77,78].

Both chronic low fat intake (less than 20% of total daily energy) and strategies promoting high fat–low CHO diets for supposed performance benefits [25] should be discouraged. Although very-low-fat vegan diets (<10% of energy from fats) have been suggested for the prevention and treatment of cardiovascular disease and diabetes [79,80,81], these diets are too restrictive for athletes undergoing intense training regimens, and incompatible with the proper intake of PRO and CHO.

### 4.5. Micronutrients

As far as micronutrients are concerned, the number of servings indicated in Table 3 can satisfy the recommended intakes, as shown in Table 4. Particular attention should be paid to the intakes of vitamin D and vitamin B12, which may occur most reliably through the regular consumption of supplements.

Vitamin D3 (cholecalciferol) is a fat-soluble vitamin that plays a vital role in many bodily functions [82]. Although its primary function is to regulate the absorption and metabolism of calcium, which is essential for strong bones and teeth, other important health issues have been related to vitamin D [83,84,85]. It can be challenging for all people, including vegetarians, to meet the recommended daily intake of vitamin D3 [86,87]. However, there are several strategies that can be used to ensure that they receive enough vitamin D, including: fortified foods (many plant-based milks, breakfast cereals, and orange juices are fortified with vitamin D, which can be a good source of the nutrient for vegans), supplements, in addition to sun exposure [49,83,84,88].

Vitamin B12 is an essential micronutrient that is not naturally found in plant foods. This vitamin is important for the formation of red blood cells and the maintenance of a healthy nervous system [89,90]. All vegetarians are recommended to consume vitamin B12 supplements and/or fortified foods such as fortified plant-based milks, breakfast cereals, and meat substitutes to meet the DRIs [2,91].

In addition to satisfying the athlete’s nutritional needs and not interfering with sports performance, well-planned vegetarian diets (i.e., lacto-ovo and vegan) can be beneficial for the athlete’s health, potentially counteracting their higher risk of ischemic heart disease (IHD) [34]; the scientific literature reports that a vegetarian diet is associated with a reduction in the risk of IHD by 25% [92], and that, in general, the health benefits are greater in vegetarians than in nonvegetarians [93].

## 5. Conclusions

By means of the VegPlate for Sports, we wanted to provide nutrition professionals with a practical tool that allows the planning of vegetarian menus to meet the nutritional needs of their clients.

The principles at the basis of the method are the same as those used for other segments of the population, i.e., the satisfaction of basal energy nutritional needs, associated with the nutritional implementation requested in specific phases of life (such as in pregnant and breastfeeding women) or certain days, i.e., as happens in the athlete, when their needs increase because they are most active.

We hope that the VegPlate for Sports, and more in general, the VegPlate method, can become a mindset for nutrition professionals, allowing them to better respond to the needs of their vegetarian patients and clients, including athletes.

This method could allow for patient or client self-management, once taught by the professional how to make their plant-based diet adequate, pleasant, and varied.

## Figures and Tables

**Table 1:** Serving sizes specific for each food group of the VegPlate for Sports (* = calcium-rich foods).

Food Group	Foods	Serving Size
(a) Grains	Bread and crackers Grain kernels (rice, barley, corn, wheat, spelt, kamut, oat, rye, millet, and quinoa) Pasta ‘’ Bulgur and cous-cous ‘’ Popcorn Ready-to-eat cereals (enriched with calcium *) Nondairy milk made from cereals (enriched with calcium **) Potatoes (if frequently consumed)	30 g 80 g (cooked); 30 g (raw) 80 g (cooked); 30 g (raw) 80 g (cooked); 30 g (raw) 30 g 30 g 200 mL 120 g
(b) Protein-rich foods	Legumes (soybeans *) ‘’ Tofu * or tempeh * Meat analogues (with soy or gluten) Soy (enriched with calcium **) or cow’s ** milk Soy (enriched with calcium **) or cow’s ** yoghurt Egg (no more than 1–2/week) Cheese*	80 g (cooked); 30 g (raw) 80 g 30 g 200 mL 125 g one 30 g
(c) Vegetables	Cooked or raw vegetable (rocket *, cabbage *, broccoli *, artichoke *, lettuce *, and endive *) Vegetable juice	100 g 100 g
(d) Fruits	Raw fruit Cooked or sliced fruit Dry fruit Fruit juice (enriched with calcium *)	150 g (one, medium) 150 g 30 g 150 mL
(e) Nuts and seeds	Nut butter (almond *) Seed butter (sesame butter, tahini *) Nuts (almonds *) or seeds (sesame *)	30 g 30 g 30 g
(f) Fats	Oil, mayonnaise, and soft margarine	5 g
(g) Calcium-rich foods	Marked with *	1 * = 1 serving, 2 ** = 2 servings
(h) Omega-3-rich foods (belonging to group e and f)	Flaxseed oil Flaxseeds, to be consumed ground Chia seeds, to be consumed ground Walnuts	5 g 10 g 15 g 30 g

**Table 2:** Mean energy and macronutrient, fiber, and water content of one average serving of each food group.

FOOD	Energy (kcal)	Protein (g)	Fats (g)	Total CHO (g)	Starch (g)	Sugars (g)	Fiber (g)	Water (g)	Iron (mg)	Calcium (mg)	Zinc (mg)
Grains	98.21	2.71	0.45	22.05	20.43	1.45	1.33	36.89	0.57	30.80	0.44
Protein-Rich	87.91	9.49	3.09	5.90	5.08	1.40	2.44	67.84	2.32	112.49	0.85
Vegetables	22.41	1.98	0.29	3.23	0.56	2.68	2.29	91.15	1.29	55.77	0.41
Fruits	63.60	1.04	0.35	15.03	0.23	14.81	2.52	122.10	0.64	33.60	0.19
Nuts and Seeds	161.33	6.23	14.56	1.71	0.87	0.73	4.96	1.81	1.65	50.05	1.77
Fats	44.58	0.00	5.00	0.00	0.00	0.00	0.00	0.00	0.01	0.03	0.00

**Table 3:** Meal Planning of the VegPlate for Sports.

	Energy (Kcal)	Grains (Servings)	Protein-Rich (Servings)	Vegetables (Servings)	Fruits (Servings)	Nuts and Seeds * (Servings)	Fats * (Servings)
50 Kg BW							
Main	1800	7	4	5	0.5	0.5	11
Pre-		2	1	0	2	0	0
Post-		1	1	0	1.5	0.5	0
Total	2500	10	6	5	4	1	11
55 Kg BW							
Main	2000	9	4	5	0	0.5	11
Pre-		3	1	0	1	0	0
Post-		0	1.5	0	3	0.5	0
Total	2700	12	6.5	5	4	1	11
60 Kg BW							
Main	2200	9.5	4	5	0.5	0.5	14
Pre-		3	1	0	1.5	0	0
Post-		0.5	1.5	0	3	0.5	0
Total	3000	13	6.5	5	5	1	14
65 Kg BW							
Main	2300	10	4	5	1	0.5	15
Pre-		3.50	1	0	1	0	0
Post-		0.50	1.5	0	3	0.5	0
Total	3200	14	6.5	5	5	1	15
70 Kg BW							
Main	2500	12	4	5	1	0.5	15
Pre-		4	1	0	1	0	0
Post-		1	1.5	0	3	0.5	0
Total	3500	17	6.5	5	5	1	15
75 Kg BW							
Main	2700	14	4	5	1	0.5	15
Pre-		4.5	1	0	1	0	0
Post-		1.00	1.5	0	3	0.5	0
Total	3700	19.5	6.5	5	5	1	15
80 Kg BW							
Main	2900	15	4	5	1.5	0.5	16
Pre-		5	1	0	0.5	0	0
Post-		1	2	0	3	0.5	0
Total	3900	21	7	5	5	1	16
85 Kg BW							
Main	3100	16	4	5	2	1	16
Pre-		5.5	1.25	0	0	0	0
Post-		2	1.75	0	3	0	0
Total	4200	23.5	7	5	5	1	16
90 Kg BW							
Main	3200	17	4	5	1.5	1	17
Pre-		6	1.5	0	0	0	0
Post-		1.5	2	0	3.5	0	0
Total	4400	24.5	7.5	5	5	1	17

* 2 servings of omega-3 rich foods should be provided by these groups.

**Table 4:** Nutritional composition of the serving patterns shown in Table 3.

	Energy	Protein	Fats	Total CHOs	Starch	Sugars	Fiber	Iron	Calcium	Zinc	Vitamin B1	Vitamin B2	Vitamin C	Vitamin B3	Vitamin B6
	kcal	g	g	g	g	g	g	mg	mg	mg	mg	mg	mg	mg	mg
50 Kg BW															
Main	1756.61	70.43	79.39	202.47	166.67	36.89	34.24	20.98	986.30	9.49	1.41	2.01	203.40	16.26	3.38
Pre-	410.04	16.99	4.69	80.07	46.39	33.91	10.15	4.73	241.38	2.11	0.38	0.43	73.49	4.23	0.94
Post-	360.69	16.88	11.34	51.35	26.29	25.43	10.03	4.67	218.81	2.47	0.35	0.39	53.46	3.21	0.78
Total	2530.34	104.27	95.41	333.89	239.35	96.22	54.42	30.42	1446.29	14.06	2.14	2.82	330.35	23.70	5.10
55 Kg BW															
Main	1921.24	75.32	80.13	239.05	207.41	32.38	35.64	21.80	1031.10	10.28	1.50	2.05	194.15	18.20	3.61
Pre-	444.65	18.65	4.80	87.08	66.60	20.55	8.95	4.66	238.58	2.36	0.38	0.40	44.12	4.81	0.95
Post-	401.83	20.48	12.95	54.80	8.74	46.89	13.71	6.22	294.66	2.74	0.45	0.56	99.45	3.38	1.02
Total	2770.72	114.43	97.87	380.94	282.75	99.82	58.30	32.72	1564.13	15.36	2.33	3.01	337.72	26.39	5.59
60 Kg BW															
Main	2136.99	77.19	95.51	257.59	217.74	40.50	37.56	22.44	1063.30	10.59	1.56	2.09	212.47	19.02	3.75
Pre-	476.45	19.18	4.98	94.60	66.71	27.95	10.21	4.98	255.38	2.46	0.41	0.43	60.62	5.07	1.02
Post-	450.94	21.83	13.18	65.83	18.95	47.62	14.37	6.50	310.05	2.96	0.48	0.58	101.26	3.93	1.09
Total	3067.38	118.18	113.65	418.02	303.40	116.07	62.15	33.97	1628.53	15.99	2.45	3.10	374.35	28.02	5.87
65 Kg BW															
Main	2262.85	79.07	100.91	276.13	228.07	48.63	39.49	23.06	1095.49	10.90	1.62	2.14	230.78	19.83	3.89
Pre-	493.75	20.01	5.03	98.11	76.81	21.27	9.62	4.95	253.98	2.58	0.41	0.42	45.94	5.36	1.03
Post-	450.94	21.83	13.18	65.83	18.95	47.62	14.37	6.50	310.05	2.96	0.48	0.58	101.26	3.93	1.09
Total	3210.54	120.88	119.10	440.07	323.83	117.52	63.48	34.55	1659.33	16.43	2.51	3.14	377.98	29.13	6.01
70 Kg BW															
Main	2459.27	84.48	101.82	320.23	268.93	51.52	42.15	24.20	1157.09	11.78	1.74	2.21	238.04	22.04	4.19
Pre-	542.86	21.36	5.26	109.13	87.03	22.00	10.28	5.23	269.38	2.80	0.44	0.44	47.75	5.92	1.10
Post-	500.04	23.18	13.41	76.85	29.17	48.34	15.04	6.79	325.45	3.18	0.51	0.60	103.08	4.48	1.17
Total	3505.18	129.00	120.46	506.21	385.12	121.86	67.47	36.27	1751.72	17.74	2.69	3.24	388.86	32.44	6.46
75 Kg BW															
Main	2655.70	89.89	102.72	364.33	309.79	54.42	44.81	25.35	1218.68	12.66	1.86	2.28	245.29	24.25	4.48
Pre-	591.97	22.71	5.48	120.15	97.24	22.72	10.95	5.52	284.77	3.02	0.47	0.46	49.56	6.47	1.18
Post-	500.04	23.18	13.41	76.85	29.17	48.34	15.04	6.79	325.45	3.18	0.51	0.60	103.08	4.48	1.17
Total	3750.71	135.76	121.60	561.34	436.20	125.48	70.79	37.70	1828.71	18.84	2.83	3.33	397.93	35.20	6.83
80 Kg BW															
Main	2830.66	93.11	108.35	393.89	330.33	63.27	47.40	26.25	1266.28	13.19	1.95	2.34	265.42	25.62	4.70
Pre-	609.27	23.55	5.54	123.66	107.34	16.04	10.35	5.49	283.37	3.14	0.47	0.45	34.88	6.76	1.18
Post-	544.00	27.93	14.95	79.80	31.71	49.04	16.26	7.95	381.70	3.60	0.58	0.72	103.20	4.97	1.36
Total	3986.93	144.56	128.82	597.36	469.38	128.35	74.01	39.73	1931.15	19.92	2.99	3.50	403.49	37.34	7.24
85 Kg BW															
Main	3041.34	99.46	116.25	424.31	351.31	72.48	52.47	27.97	1338.90	14.61	2.10	2.43	285.64	27.33	4.97
Pre-	648.56	26.75	6.37	128.64	118.72	9.71	10.36	6.03	310.09	3.48	0.50	0.49	20.25	7.29	1.29
Post-	539.57	25.15	7.35	99.52	50.43	49.77	14.50	7.12	359.35	2.94	0.54	0.67	106.67	5.49	1.36
Total	4232.46	151.33	129.95	652.48	520.46	131.96	77.33	41.16	2008.14	21.02	3.14	3.59	412.56	40.10	7.61
90 Kg BW															
Main	3152.70	101.64	121.53	438.84	371.63	66.52	52.54	28.23	1352.90	14.95	2.13	2.43	272.77	28.17	5.05
Pre-	719.64	30.47	7.36	141.14	130.20	10.78	11.64	6.90	353.61	3.92	0.57	0.57	22.12	8.08	1.45
Post-	544.24	26.69	8.07	97.49	41.60	56.80	15.71	7.73	388.87	3.03	0.58	0.74	121.42	5.44	1.44
Total	4419.58	158.78	136.95	677.48	543.43	134.11	79.88	42.90	2095.18	21.88	3.27	3.75	416.31	41.69	7.94

**Table 5:** Percentage calorie contribution of macronutrients to total calories in the VegPlate for Sports.

BW (kg)	Energy (kcal)	Protein (% kcalories)	Fats (% kcalories)	Total CHO (% kcalories)	Sugars (% kcalories)
50	2530.34	17	34	53	15
55	2770.72	17	33	56	15
60	3067.38	16	34	56	15
65	3210.54	15	33	55	15
70	3505.18	15	31	58	14
75	3750.71	15	30	61	14
80	3986.93	15	30	61	13
85	4232.46	14	28	62	13
90	4419.58	14	28	62	12

## Data Availability

Publicly available datasets were analyzed in this study. This data can be found here: [www.bda-ieo.it; fdc.nal.usda.gov].
